# Online psycho-education to the treatment of bipolar disorder: protocol of a randomized controlled trial

**DOI:** 10.1186/s12888-016-1159-0

**Published:** 2016-12-22

**Authors:** Itxaso González-Ortega, Amaia Ugarte, Sonia Ruiz de Azúa, Nuria Núñez, Marta Zubia, Sara Ponce, Patricia Casla, Josu Xabier Llano, Ángel Faria, Ana González-Pinto

**Affiliations:** 1 Center for Biomedical Research in the Mental Health Network (CIBERSAM), Department of Psychiatry, Araba University Hospital, University of the Basque Country, Olaguibel Street 29, 01004 Vitoria, Spain; 2 Centro de Investigación en Cronicidad-Kronikgune, Barakaldo, Spain; 3 IK4-Tekniker, Gipuzkoa, Spain; 4 Osatek, Bilbo, Spain; 5 Subdirección de Informática, Sistemas de Información-Osakidetza, Vitoria, Spain

**Keywords:** Bipolar disorder, Psychoeducation, Telemedicine

## Abstract

**Background:**

Bipolar disorder patients frequently present recurrent episodes and often experience subsyndromal symptoms, cognitive impairment and difficulties in functioning, with a low quality of life, illness relapses and recurrent hospitalization. Early diagnosis and appropriate intervention may play a role in preventing neuroprogression in this disorder. New technologies represent an opportunity to develop standardized psychological treatments using internet-based tools that overcome some of the limitations of face-to-face treatments, in that they are readily accessible and the timing of therapy can be tailored to user needs and availability. However, although many psychological programs are offered through the web and mobile devices for bipolar disorder, there is a lack of high quality evidence concerning their efficacy and effectiveness due to the great variability in measures and methodology used.

**Methods:**

This clinical trial is a simple-blind randomized trial within a European project to compare an internet-based intervention with treatment as usual. Bipolar disorder patients are to be included and randomly assigned to one of two groups: 1) the experimental group (tele-care support) and 2) the control group. Participants in both groups will be evaluated at baseline (pre-treatment) and post-treatment.

**Discussion:**

This study describes the design of a clinical trial based on psychoeducation intervention that may have a significant impact on both prognosis and treatment in bipolar disorder. Specifically, bringing different services together (service aggregation), it is hoped that the approach proposed will significantly increase the impact of information and communication technologies on access and adherence to treatment, quality of the service, patient safety, patient and professional satisfaction, and quality of life of patients.

**Trial registration:**

NCT02924415. Retrospectively registered 27 September 2016

**Electronic supplementary material:**

The online version of this article (doi:10.1186/s12888-016-1159-0) contains supplementary material, which is available to authorized users.

## Background

Bipolar disorder patients frequently present recurrent episodes and often experience subsyndromal symptoms, cognitive impairment and difficulties in functioning with a low quality of life [1–3], illness relapses and recurrent hospitalization [4]. The number of episodes is an important contributor to the stage progression of the illness from less to more severe presentations and consequently poor outcome. Therefore, early diagnosis and appropriate intervention may play a role in preventing neuroprogression in this disorder [5]. Pharmacological treatments such as mood stabilizers, alone or in combination with antipsychotics, appear to be effective to improve symptoms [6, 7]. However, patients often do not adhere to medication and one third of them discontinue the medication completely, increasing their number of relapses [8]. There is evidence of the efficacy of psychological interventions as an adjunct to pharmacotherapy in bipolar disorder to improve clinical and functional outcomes at follow-up [9], with better adherence [10], prevention and delay of relapse [11, 12] and an improved quality of life for patients [13].

The early stages of bipolar disorder is usually a critical period, and the time during which the disorder causes the most suffering in patients and their families, partly because of the impact the diagnosis and a lack of knowledge about the symptoms. Early educational interventions could increase knowledge and awareness of the illness and treatment adherence and thereby reduce relapses and hospitalizations [14]. Cognitive-behavioral therapy has also proven to be effective in the treatment of bipolar disorder [15, 16]. Other interventions such as interpersonal social rhythm therapy, family-focused therapy and mindfulness approaches are also indicated [9].

However there are several obstacles to the successful implementation of these types of psychological treatments. One of the major problems is the face-to-face nature of therapy that requires the patient and the therapist to be available at the same time. The level of commitment required for such treatments is difficult for many patients. Specifically, the therapy is administered over a long period of time (e.g., 4-6 months) and the sessions take place on a fixed date and at a fixed time (e.g., once a week, lasting 1 h). These aspects of the treatment are associated with a high rate of dropout, especially in more functional patients. Moreover, to implement these intervention programs specialized therapists are needed and there is a gap between availability and demand [17, 18], and hence, in many health systems, the provision of such treatments is limited. Therefore, there is a need to develop effective evidence-based interventions focused on the individual characteristics of patients allowing them to cope better with their symptoms in a way that is feasible and cost effective for healthcare systems [9, 12].

New information and communication technologies represent an opportunity to develop standardized psychological treatments using internet-based tools that overcome some of the limitations of face-to-face treatments, in that they are readily accessible and the timing of therapy can be adjusted to the need and availability of each user. Moreover, such intervention programs offer the possibility of patients self-monitoring and continuously assessing their mood (depressive and manic symptoms) as well as other variables such as sleep or physical and social functioning, with two-way feedback between patients and healthcare providers [19, 20]. This type of intervention facilitates early intervention for subsyndromal symptoms and helps patients become more aware of their illness, manage their symptoms better and improve their lifestyle and attitudes towards medication [21]. Nevertheless, although many online psychological programs for bipolar disorder are offered through the web [21–29] and mobile devices [19, 20, 30–34], there is a lack of high quality evidence about their efficacy and effectiveness due to the variability in measures and methodology used [9].

## Methods

### Objectives

#### General objective

To describe the design of an online psychoeducation treatment using new technologies and to compare its effectiveness with standard treatment in patients with bipolar disorder.

#### Specific objective

- To assess the effectiveness of a psychoeducation treatment based on telemedicine versus usual treatment in relation to outcomes in bipolar disorder (patients’ illness awareness, symptoms, functionality, relapses and rehospitalizations)
- To assess satisfaction with the treatment received and the contact with the health service

### Hypothesis

- The online treatment of bipolar disorder will be effective for increasing awareness of the illness, improving depressive and manic symptoms and functionality of patients, and reducing relapses and hospitalizations.
- Patients receiving online treatment will be more satisfied with the treatment received and their contact with the health service than patients receiving the standard treatment.

## Design

This is a clinical trial is a simple-blind randomized trial within a European project titled “The Future Internet Social and Technological Alignment Research” (FI-STAR), to compare an internet-based intervention with treatment as usual. This clinical trial fulfills with the Standard Protocol Items: Recommendations for Interventional Trials (SPIRIT Checklist) (Additional file 1).

FI-STAR provides a solution for Bipolar Patient Treatment Management (BPTM) in the form of a platform developed to support telecare of bipolar disorder patients. The BPTM platform involves clinical professionals (psychiatrists, psychologists and psychiatric nurses), patients and technicians connected using interfacing systems of the integrated multichannel service center, OSAREAN, which was already in place prior to this project. That is, the proposed solution for these patients has been developed with the internal legacy systems of Osakidetza, the largest important health service provider in the Basque Country.

The platform designed provides advanced communication channels and interaction tools to treat, monitor, support and follow-up patients with bipolar disorder. The treatment consists of online psychoeducation which includes learning sessions (with associated assessment questionnaires) and related psychotherapy exercises. The participants will be able to access the psychoeducation functionalities, whenever and from wherever they choose, using whichever of the possible interfaces (PC, tablet, or smartphone) they have and prefer. The psychiatric personnel defines the patient’s baseline parameters, assigns assessment questionnaires for patient monitoring, specifying how often they should be completed, and sets evaluation rules to generate alerts. Both psychiatric personnel and patients should be able to view follow-up data (progress through the educational material, patients’ notes, changes in monitoring parameters, reported medication intake and any side effects, event log, and patient alerts/notifications).

### Participants

Bipolar disorder patients who meet the inclusion criteria are to be included and randomly assigned to one of two treatment groups. To achieve a power of 80% to detect differences from the null hypothesis, H0: μ1 = μ2, using a bilateral Student’s t test for two independent samples, with a significance level of 5%, 25 patients will be included in the experimental group (tele-care support) and 25 patients in the control group, summing to a total of 50 patients for the study.

### Inclusion criteria

- Having a diagnosis of bipolar disorder
- Being aged between 18 and 50 years
- Being in remission (in a euthymic state)
- Being familiar with current information and communication technologies (computer, tablet and/or smartphone)
- Being under treatment in the mental health network of the Basque Country (Osakidetza)

### Exclusion criteria

- Having hearing or literacy difficulties
- Having a diagnosis of mental retardation
- Not having access to a smartphone or other suitable device
- Having manic or depressive symptomatology at the time of inclusion (Hamilton Depression Rating Scale [35, 36] > 16 and Young Mania Rating Scale (YMRS) [37, 38] > 8)
- Having suicidal ideation

### Randomization

Patients are to be randomly assigned to either a control or an intervention group by permuted block randomization with a block size of 4 and a 1:1 allocation using a computer-generated random sequence. The allocation sequence will be prepared by an independent person not otherwise involved in the trial. The control group will receive the standard treatment (pharmacotherapy and regular sessions with their psychiatrist) and the intervention group will receive an online psychoeducation treatment, in addition to pharmacological treatment prescribed by the psychiatrist.

### Assessment

**Fig. 1:**
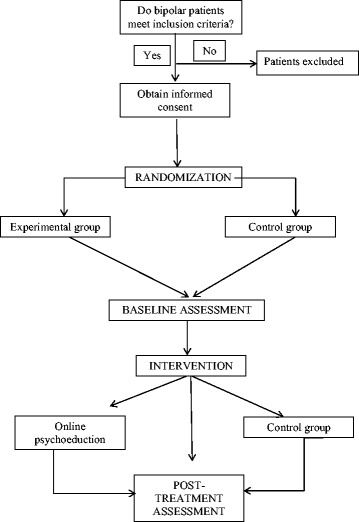
Study procedure

Patients will be assessed after being informed of the objectives of the study and giving their informed consent to participate. Data will be collected following an assessment that will be implemented at baseline and post-treatment (Fig. 1).

Patients are diagnosed according to the revised fourth edition of the Diagnostic and Statistical Manual of Mental Disorders (DSM-IV-TR) criteria using the Structured Clinical Interview for DSM-IV-TR, Axis I Disorders [39]. These interviews are carried out independently by two experienced clinicians.

Patients are also assessed with the Mood Disorder Questionnaire [40, 41]. This instrument is a self-report screening test for mood disorders derived from the DSM-IV-TR criteria designed to assess symptoms of bipolar spectrum disorder.

All patients are to be assessed at baseline and post-treatment, in terms of depressive, manic and anxiety symptomatology, psychosocial functioning and lifestyle habits: diet, physical activity and sleep.

Depressive symptoms are measured with the Beck Depression Inventory (BDI-II) [39, 40] and the Beck Hopelessness Scale (BHS) [42, 43]. The BDI-II is a 21-question multiple-choice self-report inventory, one of the most widely used psychometric tests for measuring the severity of depression [39, 40].

The BHS is a 20-item self-report inventory designed to measure three major aspects of hopelessness: feelings about the future, loss of motivation, and expectation [44, 45]. Manic symptoms are assessed with the YMRS [37, 38], an 11-item scale used to assess the severity of this type of symptoms.

Anxiety symptoms are measured with the State-Trait Anxiety Inventory (STAI) [46]. The STAI is composed of 40 items with two subscales: the State Anxiety Scale (S-Anxiety) evaluates the current state of anxiety and the Trait Anxiety Scale (T-Anxiety) evaluates relatively stable aspects of “anxiety proneness”.

The functioning of patients is measured using the Functioning Assessment Short Test [47]. This is a brief instrument designed to assess functional impairment in severe mental disorders. The 24 items of the scale cover 6 specific areas of functioning: autonomy, occupational functioning, cognitive functioning, financial issues, interpersonal relationships, and leisure time.

In addition, all patients are monitored daily by collecting data on the following variables: mind state, number of hours slept, presence of stressful events, adherence and side effects and alcohol and drug use.

### Intervention program

The intervention program consists of nine sessions of online psychoeducation (once a week) focused on patient empowerment by providing specific telecare capabilities and multi-channel communication to enable interaction between the patients and the healthcare professionals (psychiatrists, psychologists and psychiatric nurses).

The psychoeducation comprises learning sessions (with associated assessment questionnaires) and related psychotherapy exercises using their preferred interface (i.e., PC, tablet, or smartphone).

The participants will be able to see the learning status of each assigned learning session and access the related multimedia content. It will be possible to download the associated PDF documents, if participants want, but the video content will only be available online. They will be able to annotate material for the learning sessions and store these notes for future consultation. Once participants have worked through the material for a given learning session, they will have to complete and submit, online, a specific questionnaire to assess their level of understanding.

If the submission process fails, the system should provide appropriate feedback (process failure - subsequent delivery attempt) to the user. If the process succeeds, the system will evaluate the results of the assessment questionnaire, store them along with the usage parameters, provide feedback to the user on the success of the submission process and on the results of the questionnaire, and update the learning status of the learning session (depending on whether they answered the questions correctly). The data collected will be stored in the BPTM environment.

The psychotherapy exercises are ongoing activities that require previous knowledge from the related learning sessions. There are a range of types of exercises on topics such as symptom detection and relapse prevention. Once the related learning session has been successfully completed, the patients will be able to access the related psychotherapy exercises and update them. As it is an ongoing process, the patients will be able to access the psychotherapy exercises over time. The new updates will be sent and stored and the user will get feedback on the status of the data submission process (successful or not). Like for the learning sessions, the data collected will be stored in the BPTM environment.

If clarification is required in relation to learning sessions or psychotherapy exercises, the participants will be able to interact with the psychiatric personnel through asynchronous messages. The user will get feedback on the status of the submission process (successful or not) and the messages will be stored in the BPTM environment.

The system will also be able to, automatically, identify potential alerts. Some examples of alert triggers are: a patient not having started the psychoeducation process after a certain period of time, and a patient “failing” the assessment questionnaire more than once. Patients should receive notifications based on their preferences (e.g., call center call, SMS, instant messaging, or email – to be decided). Alerts handled by the call center will be managed through the OSAREAN platform.

The content of the nine online psychoeducation sessions is as follows:

- Session 1. *What is bipolar disorder*?
  Information is provided to the patient about the nature of bipolar disorder (a description of the symptoms that characterize the disorder and biological mechanisms involved).
- Session 2. *Symptoms of the disorder*
  The patient is taught to identify the characteristic symptoms of each phase of bipolar disorder.
- Session 3. *Course and prognosis of the disorder*
  Risk factors that may contribute to worsening the course of the disorder are explained, as are factors related to a good prognosis.
- Session 4. *Pharmacological treatment for the disorder*
  The mechanisms of action of medications are explained, as are potential side effects of the medication, in order to improve adherence and prevent the patient stopping the medication.
- Session 5. *Relapse prevention*: *risk and protective factors*
  The patient is taught to recognize the symptoms that precede a relapse and risk factors that increase the likelihood of relapse, in order to improve the course of their illness.
- Session 6. *What can be done about a relapse*?
  It is explained to the patient that rapid identification of the prodromal symptoms and timely intervention can help to prevent relapse and bring him/her back to normal.
- Session 7. *Healthy lifestyle*
  Tips to maintain a healthy lifestyle are provided, seeking to improve the health, quality of life, satisfaction and happiness of the patient.
- Session 8. *Toxic habits*
  The effect of toxic substances on the illness is explained, to motivate the patient to avoid their use, and thereby improve the prognosis of the illness.
- Session 9. *What can I do to improve my condition*?
  The patient is taught how to take an active role in their illness to improve their health and quality of life.

### Statistical analysis

Outcomes in each of the groups of patients will be analyzed, to assess whether there has been clinical and/or functional improvement, comparing between baseline and post-treatment by T Student-test for differences in the means of related samples.

The influence of the treatment will be analyzed comparing the clinical and functional improvement in each group through an analysis of covariance (ANCOVA) using the baseline and post-treatment scores.

Finally, logistic regression models will be built to analyze variables potentially associated with relapses and hospitalizations such as sleep, lifestyle, medication adherence, side effects and adverse events.

## Discussion

Telemedicine is potentially useful to complement usual treatment, helping patients to manage their illnesses, and helping health providers improve the care delivered, especially by giving clinicians feedback. Nevertheless, few randomized clinical trials have shown the effectiveness of this approach in bipolar disorder. The randomized clinical trial described in this paper concerns an internet-based psychoeducation program for bipolar disorder that could have a significant impact on both prognosis and treatment, and may be useful for identifying patients who need earlier and/or continuous therapeutic interventions.

The results of our study may have a significant impact on prognosis of bipolar disorder patients. Below, we set out what our study seeks to accomplish and how:

- To help healthcare professionals care for as many patients as possible and save time (for both professionals and patients) as well as to collect data for research, the BPTM platform will increase the availability of psychological treatment by providing tele-services (cheaply and with easy access), increase the therapist’s efficiency, reduce the need for face-to-face consultations, allow the involvement of the call center nurses to response promptly to alerts (e.g., by calling patients and/or caregivers) and facilitate patient handover between doctors.
- To enhance the quality of care, improve the course of the illness, and reduce costs to the healthcare system, the BPTM platform will help clinicians to improve patient follow-up (i.e., achieve closer monitoring of symptoms, and more frequent contact with patients including online consultations), support early identification of risk factors to respond better to patient needs (thereby, reducing relapses, hospitalizations and hospital bed days, and related costs) and provide an additional way to interact with both patients and caregivers (namely, through secure asynchronous messaging).
- To supply online treatment suited to each patient’s needs, the BPTM platform will allow psychiatric personnel to tailor patient’s treatment (e.g., assignation of learning sessions and questionnaires to be completed by given users, and defining individual patient’s baseline parameters to tune the rules to generate alerts).
- To provide patients with a way to enhance their knowledge about the management of their own illness and strategies to minimize its consequences (in particular, to reduce psychotic, anxious, manic and depressive symptoms) and to increase their treatment adherence, the BPTM platform will provide access to psychoeducation and psychotherapy in the form of learning sessions (including multimedia content such as video tutorials, presentations, PDF documents and optionally audio) and specific psychotherapy exercises which can be performed wherever and whenever the patient chooses (i.e., ensuring the availability of care for busy patients).
- To enable patients to be actively involved in their treatment process, the BPTM platform will provide a way to monitor their status remotely by enabling the collection of data on physiological/biometric parameters (either manually or automatically using sensors) and psychological parameters and images (e.g., photographs to identify skin problems), medication intake, and potential medication side effects as well as the course of the illness, by interacting with the system through their preferred available interface (PC, tablet or smartphone, users being asked to utilize devices they already have available, thereby reducing costs and promoting the sustainability of the service). Optionally biometric data could be gathered automatically through mobile devices by connecting to specific biometric sensors (e.g., activity monitors, pulse oximeters, body composition monitors).
- To allow caregivers to become more involved, increase their knowledge about bipolar disorder and interact with clinicians to be able to better support the care recipients, the BPTM platform will enable their access to specific psychoeducation-related materials and will provide a channel of communication to interact with the psychiatric personnel. It is envisaged that the involvement of caregivers and/or relatives in the treatment will reduce the patient’s acute symptoms by stopping them developing and providing support for early detection. Patient adherence to treatment may also be improved by providing a better family environment and reducing patient anxiety caused by family disputes.
- To ensure safety, confidentially and privacy, the BPTM platform will comply with all local regulations and implement protocols to safeguard the confidentiality of medical data exchanged.
- To supply user-friendly interfaces and facilitate personalized interaction, the BPTM platform will allow participants (i.e. patients and caregivers) to use their preferred interface (i.e. PC, tablet or smartphone, as available) and have a common user experience regardless of the choice of device, and allow them to select their preferred channels of communication for notifications. The platform will also support well-known interaction metaphors and usability criteria.

The limitations of this study include difficulties in the management of the new technologies by patients, especially the older ones or those with more cognitive impairment. Likewise, the absence of face-to-face modality in these types of interventions may decrease adherence to the treatment due to a potential weakening of the therapeutic relationship with the therapist and loss of associated benefits. Nevertheless, combined approaches can be implemented, such as using the internet-based platform with additional support from the therapist for some patients.

In conclusion, the platform we propose may help to improve functionality and quality of life in bipolar disorder, and what is most important, the act of bringing different services together (service aggregation) might significantly increase the impact of information and communication technologies on access and adherence to treatment, the quality of the service, patient safety, patient and professional satisfaction, and quality of life of the patients.

## Additional file

Additional file 1: SPIRIT 2013 Checklist. Recommended items to address in a clinical trial protocol and related documents* (DOC 122 kb)

## Abbreviations

BDI-II: Beck depression inventory
BHS: Beck hopelessness scale
BPTM: Bipolar patient treatment management
DSM-IV-TR: Diagnostic and statistical manual of mental disorders
FI-STAR: The future internet social and technological alignment research
STAI: State-trait anxiety inventory
